# Effects of Rule Changes on Game-Related Statistics in Men’s Water Polo Matches

**DOI:** 10.3390/sports5040084

**Published:** 2017-11-01

**Authors:** Joaquin Madera, Victor Tella, Jose M. Saavedra

**Affiliations:** 1 Department of Physical Education and Sport, University of Valencia, 46010 Valencia, Spain; tella@uv.es; 2 Physical Activity, Physical Education, Health and Sport Research Centre (PAPESH), Sport Sciences Department, School of Science and Engineering, Reykjavik University, 101 Reykjavik, Iceland; saavedra@ru.is

**Keywords:** notational analysis, performance, match outcome

## Abstract

The aim of this study was to investigate the effects of rule changes on game-related statistics in men’s water polo matches. A total of 856 men’s matches played in all Olympic Games and World Championship since 1936 was analysed. The game-related statistics considered were: total goals, winners’ goals, losers’ goals, goals per minute, goals difference, relationship difference goals and total goals, and relationship difference goals and winners’ goals. The rule changes were grouped by structural (game and period) and functional changes (possession time, exclusion time, timing, minimum distance to take a direct shot). Differences between rule changes were determined using a one-way ANOVA. In general, the changes in water polo rules were shown to have an effect on the final result of the matches. There were differences in each rule change of duration (increased total goals and winners’ goals), period (increased total goals and winners’ goals), possession time (increased losers’ goals), timing (increased total, winners’, losers’, and decreased % goals and total goals) and fouls (increased total goals and losers’ goals). The analysis in game-related statistics through the rule changes could be used to evaluate their effects and/or justify future modifications.

## 1. Introduction

Effects of rule changes in team sports are rarely researched [1]. Water polo has shown relevant rule changes since its beginning as the first team sport at the Olympic Games in Paris 1900 [2,3]. Water polo has a large following in Europe, but it is undergoing rapid growth in the United States and Australia [4]. Generally, the rules of sport affect both structural or quantitative (i.e., space, time, equipment, and number of players) and functional or qualitative (the player’s use of structural elements) aspects [5]. Thus, water polo rules are grouped in structural (match length, period length, and field dimensions) and in functional aspects (possession time, exclusion time, timing, and fouls, among others). Since 2005, water polo structural rules have established a match length of 32 min (four periods, eight minutes each) with two minutes of rest between the first and second periods and between the third and fourth periods, and with five minutes of rest between the second and third periods, with seven players on the 30 × 25 m field. This structure has evolved from a duration time of 20 min in the first competitions to the 32 min of today’s matches, with a wider distribution in periods (i.e., two periods of 10 min, four periods of five minutes, four periods of seven minutes, and, finally, four periods of eight minutes). Similarly, the field has expanded from 30 × 20 m to 30 × 25 m. Among others, water polo’s functional rules have changed to allow a non-static timing, temporal exclusions for major fouls have been reduced (i.e., from 1 min in 1971 to 20 s today), and shooting directly from a foul (5 m today) [6]. Furthermore, the time in possession of the ball has evolved from not having a defined duration, to showing a progressive reduction (45 s, 35 s, and 30 s) [6]. Table 1 shows the main changes of water polo rules through history.

These rule changes are applied in order to improve performance (i.e., injuries, physiological variables, and game statistics), to attract spectators and to address commercial interests or to adapt the sport to new practitioners or to avoid injuries [5]. However, only a few studies have analysed how the changes affect the water polo game. The study by Platanou et al. [7] showed a higher number of shots (18%), total goals (18%), goals from the centre position (38%), and a lower time in possession (17 s) with the new rules (2005). These variations encouraged a more offensive style of play [5]. Nevertheless, most of the studies analyse the relation between the number of shots and the number of goals scored [8,9,10]. Today, following these changes, the final ranking of a water polo championships shows a high percentage of predictability (83%), and it is superior to other sports like basketball, handball, volleyball, or soccer (from 73% to 76%) [11]. The changes in rules could increase equality among teams and it could allow a reduction in predictability as well as an improvement in media visibility [12]. On the other hand, these rule changes bring significant alterations to the game of water polo, an example being the increase in possession time and swimming pool dimensions. This suggests a need for greater cardiovascular endurance to maintain the ability to execute skills such as defensive postures. Similarly, a decrease in possession time suggests a need for quicker circulation of the ball, and thus the anaerobic system being more closely involved in-game. As a result, due to the lack of studies into the effects of rule changes among men’s water polo, the aim of this current study was to analyse the effects of the aforementioned rules (structural and functional) on game-related statistics in elite water polo matches.

## 2. Materials and Methods

### 2.1. Participants

The game-related statistics of 856 preliminary round men’s matches played in 856 International Championships were analysed (all Olympic Games since 1936, *n* = 465; and all World Championship games, *n* = 391). The data were retrieved from the box scores from official Olympic reports (https://www.olympic.org/) and the official OMEGA Timing Website (http://www.omegatiming.com/). The data were retrieved by one of the authors (J.M.), and entered manually into an Excel file. It was then subjected to a random check by another of the authors (J.M.S.) in order to detect possible errors. The analysis of public data taken from Websites is habitual in the field of water polo in particular [8,13,14], and of water sports in general [15,16]. Approval for this study was obtained from the Human Ethics Committee of the Universidad de Valencia.

### 2.2. Procedures

The game-related statistics (dependent variables) considered were: total goals (the sum of losers’ goals and winners’ goals or the sum of winners’ and losers’ goals), winners’ goals (total goals scored by winners), losers’ goals (total goals scored by losers), goals per minute (number of total goals of the match divided by the total duration of the match in minutes), goal difference (subtraction of losers’ goals from winners’ goals), relationship difference goals and total goals (% of difference goals of the goals scored by winners), and relationship difference goals and winners’ goals (% of difference goals of the total goals of the match). The independent variables were grouped by structural and functional rules [6]. The structural rules studied were: duration (total game/match length in minutes), period (number of periods of a match). The functional rules studied were: possession time (time for a team to score in seconds), exclusion time (time of a player that has been excluded to return to the swimming pool in seconds), timing (allowed attitude to the players when a foul has been committed), minimum distance to do a direct shot (shortest distance from where a free throw may be directly shot to the opponents’ goal).

### 2.3. Statistical Analysis

Basic statistical descriptors (mean and standard deviation) were calculated by each game-related statistic (dependent variables) in function spatial-temporal and game variables (independent variables). The normality of the data was confirmed by a Kolmogorov-Smirnov test. A one-way ANOVA was used to establish the differences by game-related statistics in function spatial-temporal and game variables (independent variables). A Bonferroni post hoc test was performed for cases where there were three or more groups in independent variables. All analyses were performed using the computer software Statistical Package for Social Sciences version 19.0 (SPSS Inc., Chicago, IL, USA).

## 3. Results

Table 2 shows the differences in the evolution of the structural variables regarding the main changes in water polo rules. There were differences in all variables with the changes in duration and period, but only in total goals and winners’ goals were the differences presented in each rule change.

The functional variables data and their relation to the evolution of game rules are shown in Table 3. In general, there were differences in all variables with the changes in possession time, exclusion time, timing, and fouls and, in particular, there were differences in each rule change of possession time (losers’ goals), timing (all variables except goal difference), and fouls (total goals and losers’ goals).

## 4. Discussion

This is the first study to analyze the differences of game-related statistics in relation to changes of the rules. In general, the changes in water polo rules showed differences in the final result of the matches. In particular, there were differences in each rule change of duration (total goals and winners’ goals), period (total goals and winners’ goals) (Table 2), possession time (losers’ goals), timing (all variables except goals difference), and fouls (total goals and losers’ goals) (Table 2). As with most studies of sports, game statistics vary after change. This study aims to understand the effects of such rule changes and to justify potential changes in the future. Concurrently, the study could allow coaches to better adapt to prospective changes and learn how those changes may affect the structure and outcome of the game.

### 4.1. Structural Changes

Duration and period were the analysed structural changes in a water polo match, which showed a duration increase from 20 min (before 1942) to 28 min (1981) and finally the current 32 min (after 2005). This increase results from the increment of the number of periods or the length of each period (Table 1). The results showed a larger number of total goals and winners’ goals. However, losers’ goals did not change when increasing the length of a match from 28 to 32 min. This seems to indicate that the losing teams did not adapt as well to these changes in the duration of the game. Even though the length of the game was increased by 4 min, the amount of goals scored by the losing teams did not increase. In addition, a study showed an increase in total goals during a match where the total length of match had been enlarged [7]. On the other side, total goals’ difference, goals’ difference/total goals ratio, and goals’ difference/winners’ goals ratio only showed increases when the length of the match increased from 20 to 28 min. So, there was no improvement of the effectiveness when increasing the length from 28 to 32 min. This variable has been studied in other works [13,17] as a predictor of the final result. Nevertheless, those studies define effectiveness as the percentage of the number of goals within the total number of shots (goals/shots × 100).

The changes regarding the period variable were analysed in two phases. The first one (up to 1949) occurred when the number of periods was increased from two to four, but by reducing the length of each one from 10 to 5 min, the total length of the match was not modified. The second change kept the number of periods and increased their length, consequently increasing the length of the match (Table 1). Regarding the first phase, total goals, winners’ goals, losers’ goals, and goals per minute increased their values. This increment is similar to other team sports (i.e., basketball) when the number of periods per match was increased [18]. This increment is related to an increment of the game intensity [19,20] and improvement in the coach-player communication between periods [21]. Also, winners’ goals and difference goals/total goals values became higher in both winners’ and losers’ teams, thereby reducing the predictability in the final result of the match [11]. In the second phase, the increase of the total length of all four periods also allowed increases in total goal, winners’ goals, goals per minute, and difference goals/total goals. Total goals and winners’ goals increased progressively when changing from five- to seven- and finally to the current eight-minute periods. Winners’ goals and difference goals/total goals only showed increases when periods changed from five to seven minutes. Thus, it is evident that the last increment (seven to eight minutes) has not modified the effectiveness as a discriminant parameter in winning teams as it was observed in previous studies [8,11,13,17].

### 4.2. Functional Changes

Regarding the possession time, results show that the stages of reduction in the variables of total goals, winners’ goals, losers’ goals, and goals per minute have increased in value. Several studies [8,14,22] showed that when the maximum possession time is set to 30 s, teams have possession on approximately 40 occasions per match with an average duration of 16 seconds. This decrease in possession time tallies with studies in other sports (i.e., basketball) where this reduction would speed up the offensive play [18]. Thus, winners’ goals and difference goals/total goals increased when the temporal limit of attack time was reduced, allowing a reduction in the predictability of the final result and an improvement in the sport’s media visibility [12].

On the other hand, changes in exclusion time produced an increase of total goals, winners’ goals, and losers’ goals. This trend was reversed with the latest reduction of the rule 35 vs. 20 s, where a decrease in total goals and winners’ goals was revealed. A previous study showed an approximate 40% effectiveness (percentage of goals scored during extra player situations relative to the number of shots made in this situation) in such circumstances [23]. Similarly, an approximate effectiveness of three goals per game in international championships has been reported [23,24]. Regarding goals per minute, a progressive increase in each phase was detected (with the exception of 2002), to the extent that this rule reduces the time of exclusion. The extra-man shows a percentage effectiveness between 40% and 50% [8,13]; future changes in the rules of exclusion should be studied so as to prevent compromising their effectiveness.

In the early years of water polo, the timing was regulated so that the players had to remain immobile between signaling a foul and restarting the match (i.e., static). This was modified in 1949 to allow the players to continue their offensive and defensive displacements (i.e., non-static). The results showed that when timing is non-static, all total goals, winners’ goals, losers’ goals, and goals per minute increase and the matches display a higher equality (i.e., goal difference). There is no doubt that non-static situations have allowed water polo to become a more dynamic sport, and this feature could result in more media interest [12] and lower match predictability [11]. Finally, the last feature of this analysis is the minimum distance to take a direct shot. This distance had been stable (i.e., 4 m) until the changes in 1996 (i.e., 7 m) and from 2005, when the minimum distance for a direct shot from a foul changed to 5 m. These changes triggered an increase in total goals, winners’ goals, and losers’ goals at each stage. Regarding the effectiveness of this shot, 16% of successful shots were calculated for male and female national teams when the minimum distance to shoot after a foul was 7 m [24]. However, a more recent study by Escalante et al. [8] showed an effectiveness of 19% in a mixed sample of male and female national teams during the 5 m rule to shoot after a foul. This seems to indicate that the decision of reducing the minimum distance to shoot from 7 to 5 m has improved the effectiveness of these types of shots.

### 4.3. Limitations

The main limitation of this study is that it only analyzes the internal logic of this sport. A water polo match is a complex phenomenon whose final result is affected by the players’, teams’, and matches’ characteristics [21]. Matches are determined by internal (studied variables) and external aspects [5]. The latter (i.e., type of tournament, scoring system, characteristics of the material, among others) have not been studied. A second limitation is that technical and tactical skill learning and execution are not considered, which may alter the results.

## 5. Conclusions

Structural and functional changes have induced changes in all game-related statistics studied, especially in total goals and winners’ goals. In this regard, the game’s own evolution has tended to a higher total time length, while the functional changes have triggered a higher game intensity. This could be taken into account by coaches when planning training sessions. To give an example, the latest modifications to the time of exclusion have led to a reduction in the total number of goals, winning goals, and goals per minute. This should alert coaches to develop their gameplay during periods of power-play by speeding up offensive play so as to not reduce effectiveness. This could be achieved through either physical or technical-tactical training. In addition, it is important to consider previous studies that can help justify the application of rule changes and their effect on improvements to the sport. This includes improving performance levels, effectiveness, predictability, and media visibility [5,11,12]. These studies help to reduce undesired effects on the sport of water polo. This is especially important if or when FINA (Fédération Internationale de Natation) are contemplating future rule changes.

## Figures and Tables

**Table 1:** Main changes of water polo rules from 1942 to 2005 [2].

Year of Change	Structural Rules		Functional Rules			
	Period × Time (Game)	Swimming Pool Dimensions	Possession Time (s)	Exclusion Time (s)	Timing	Minimum Distance (Direct Shot)
≤1942	2 × 10 min (20 min)	30 × 20 m			Static	4 m
1949	4 × 5 min (20 min)				**No static**	
1961						
1971			45	60		
1977			35	45		
1981	4 × 7 min (28 min)					
1986				35		
1991				**20**		
1996						7 m
≥2005	**4 × 8 min (32 min)**	**30 × 25 m**	**30**			**5 m**

Current rules in bold.

**Table 2:** Mean (SD) and differences (*p* < 0.05) of each variable according to duration and period (structural rules).

Variable	Duration				Period				
	20 min	28 min	32 min	Diff.	2 × 10 min	4 × 5 min	4 × 7 min	4 × 8 min	Diff.
	(a)	(b)	(c)		(a)	(b)	(c)	(d)	
	Mean (SD)	Mean (SD)	Mean (SD)		Mean (SD)	Mean (SD)	Mean (SD)	Mean (SD)	
Total goals (856)	9.53 (3.53)	16.57 (4.73)	18.17 (4.56)	a < b < c	7.72 (2.99)	11.23 (4.38)	16.57 (4.73)	18.07 (4.60)	a < b < c < d
Winners’ goals (n)	6.76 (2.96)	10.78 (4.12)	11.95 (3.96)	a < b < c	5.96 (2.88)	7.63 (3.38)	10.78 (4.12)	11.92 (3.97)	a< b < c < d
Losers’ goals (n)	2.77 (1.77)	5.78 (2,45)	6.22 (2.55)	a < b,c	1.75 (1.22)	3.59 (2.16)	5.78 (2.45)	6.15 (2.51)	a < b < c,d
Goals difference (n)	3.98 (3.36)	5.00 (4.86)	5.73 (4.81)	a < b,c	4.21 (3.26)	4.04 (3.61)	5.00 (4.86)	5.76 (4.81)	a,b < d
Goals per minute (n)	0.48 (0.18)	0.59 (0.17)	0.57 (0.14)	a < b,c	0.39 (0.15)	0.53 (0.17)	0.59 (0.17)	0.56 (0.14)	a < b <c,d
Difference goals and total goals (%)	41.70 (30.34)	29.16 (23.71)	31.03 (23.64)	a > b,c	51.41 (31.74)	36.32 (28.34)	29.16 (23.71)	31.36 (23.49)	a > b >c,d
Difference goals and winners’ goals (%)	52.48 (30.45)	40.41 (26.30)	43.23 (25.84)	a > b,c	61.65 (30.74)	48.16 (29.40)	40.41 (26.30)	43.23 (25.84)	a > b >c,d

**Table 3:** Mean (SD) and differences (*p* < 0.05) of each variable according to possession time, exclusion time, timing, and fouls (functional rules).

Variable	Possession Time					Exclusion Time							Timing			Fouls			
	No Limit	45 s	35 s	30 s	Diff.	Penalty	3 Fouls-Penalty	60 s	45 s	35 s	20 s	Diff.	Static	Non-Static	Diff.	4 m	7 m	5 m	Diff.
	(a)	(b)	(c)	(d)		(a)	(b)	(c)	(d)	(e)	(f)		(a)	(b)		(a)	(b)	(c)	
	Mean (SD)	Mean (SD)	Mean (SD)	Mean (SD)		Mean (SD)	Mean (SD)	Mean (SD)	Mean (SD)	Mean (SD)	Mean (SD)		Mean (SD)	Mean (SD)		Mean (SD)	Mean (SD)	Mean (SD)	
Total goals (856)	8.78 (3.59)	10.51 (3.45)	15.87 (4.99)	18.17 (4.60)	a < b< c < d	7.72 (3.01)	11.31 (3.62)	10.51 (3.45)	14.84 (5.70)	19.22 (4.92)	16.68 (4.64)	a < b,c < d< e > f	6.88 (3.34)	14.50 (5.58)	a < b	12.73 (5.75)	14.96 (4.03)	18.17 (4.60)	a < b < c
Winners’ goals (n)	6.55 (3.06)	7.17 (3.00)	10.33 (4.18)	11.95 (3.96)	a,b < c < d	5.94 (2.84)	8.00 (3.13)	7.17 (3.00)	9.63 (4.35)	12.83 (4.31)	10.88 (4.01)	a < b,d,e,f; b < e,f; c < d < e > f	5.83 (3.55)	9.63 (4.26)	a < b	8.58 (4.26)	9.90 (3.53)	11.95 (3.96)	a < b,c
Losers’ goals (n)	2.23 (1.65)	3.34 (1.73)	5.54 (2.48)	6.22 (2.55)	a < b < c < d	1.78 (1.27)	3.31 (1.96)	3.34 (1.73)	5.21 (2.80)	6.39 (2.60)	5.80 (2.41)	a < b,c < d < e,f	1.04 (1.00)	4.86 (2.67)	a < b	4.16 (2.73)	5.05 (2.10)	6.22 (2.55)	a < b < c
Goals difference (n)	4.32 (3.37)	3.83 (3.49)	4.79 (4.72)	5.73 (4.81)	a,b < d	4.16 (3.20)	4.69 (3.76)	3.83 (3.49)	4.42 (4.59)	6.44 (5.14)	5.08 (4.73)	a,c < e	4.79 (4.00)	4.77 (4.41)	n.s.	4.42 (4.26)	4.85 (4.19)	5.73 (4.81)	a < c
Goals per minute (n)	0.44 (0.18)	0.53 (0.17)	0.58 (0.17)	0.57 (0.14)	a < b < c,d	0.39 (0.15)	0.57 (0.18)	0.53 (0.17)	0.59 (0.17)	0.69 (0.18)	0.56 (0.15)	a < b,c,d,e,f; b,c <e; d < e > f	0.34 (0.17)	0.55 (0.17)	a < b	0.54 (0.19)	0.53 (0.14)	0.57 (0.14)	n.s.
Difference goals and total goals (%)	48.50 (31.56)	35.69 (27.94)	29.32 (23.83)	31.03 (23.64)	a >b,c,d	51.29 (31.73)	41.84 (30.44)	35.69 (27.94)	29.33 (24.51)	32.71 (24.95)	29.61 (23.45)	a >c,d,e,f; b > f	60.89 (37.70)	33.46 (26.22)	a > b	36.19 (28.86)	31.38 (23.32)	31.03 (23.64)	n.s.
Difference goals and winners’ goals (%)	58.95 (30.77)	46.83 (28.86)	40.56 (26.43)	43.23 (25.84)	a > b,c,d	61.63 (30.28)	52.54 (31.28)	46.83 (28.86)	40.33 (27.20)	44.31 (27.39)	41.16 (25.90)	a > c,d,e,f	67.94 (34.98)	44.90 (27.93)	a > b	46.95 (29.78)	43.28 (25.84)	43.23 (25.84)	n.s.

n.s. = no significance.
